# The effect of amputation level on patient mental and psychological health, prospective observational cohort study

**DOI:** 10.1016/j.amsu.2022.104864

**Published:** 2022-11-12

**Authors:** A. Abdel Rahim, A. Tam, M. Holmes, D. Mittapalli

**Affiliations:** Department of Vascular Surgery, University Hospitals Plymouth NHS Trust, United Kingdom

**Keywords:** Non-traumatic amputations, Trans-tibial amputations, Tans-femoral amputation, Hospital anxiety and depression score for anxiety (HADS-A), Hospital anxiety and depression score for depression (HADS-D), Mental and psychological health

## Abstract

**Background:**

Non-traumatic lower limb amputation is a commonly performed surgical procedure and is associated with a high prevalence of psychological morbidity including anxiety and depression. Many risk factors have been identified, including the indication for amputation, perioperative pain and sociodemographic factors.

**Objective:**

The aim of this study was to identify whether level of amputation has an impact on this psychological morbidity.

**Methods:**

A prospective observational study was conducted in a tertiary vascular unit including all adult non-traumatic amputations performed during a 6 month period. The Hospital Anxiety and Depression Scale (HADS) was used to score anxiety and depression pre and postoperatively.

**Results:**

49 patients met the inclusion criteria (22 trans-femoral amputations (AKA) and 27 trans-tibial amputations (BKA)). HADS scores for anxiety and depression were high in both groups both pre and postoperatively. A higher level of anxiety was noted in the BKA group, significantly decreasing postoperatively (p < 0.05). No other statistically significant differences were found between the two groups.

**Conclusion:**

In non-traumatic amputations, there appears to be a higher rate of pre-operative anxiety in patients undergoing trans-tibial amputation compared with trans-femoral amputees. However, the level of amputation does not appear to have a significant effect on psychological status of patients with high rates of depression and anxiety demonstrated in both groups.

## Highlights

• Part of the rehabilitation process of the non-traumatic amputee is their mental and psychological health.
• In non-traumatic amputations, there appears to be a higher rate of pre-operative anxiety in patients undergoing trans-tibial amputation compared with trans-femoral amputees.
• The level of amputation does not appear to have a significant effect on psychological status of patients.
• The cohort showed high rates of preoperative anxiety and postoperative depression.

## 1 Introduction

**Fig. 1:**
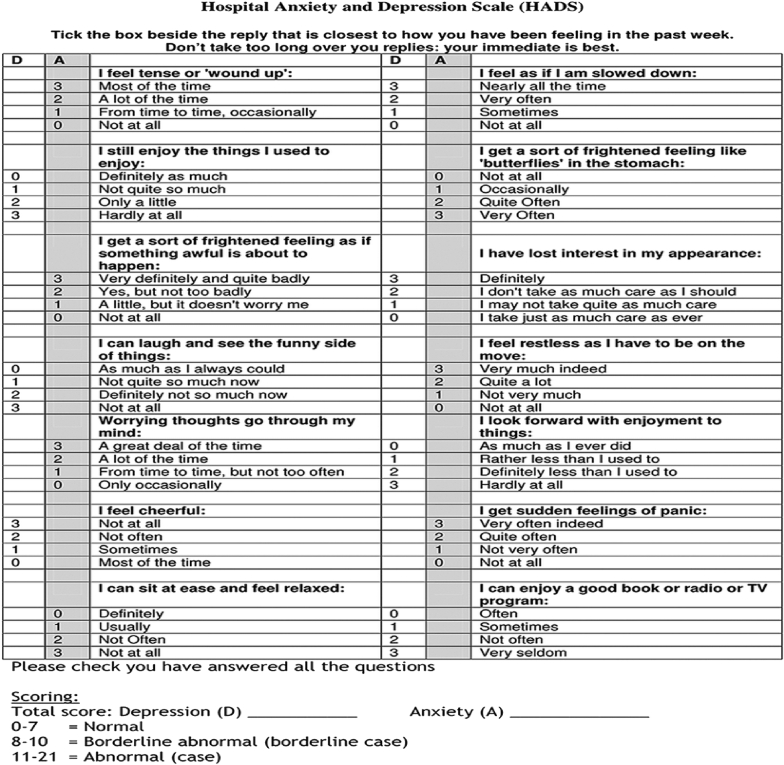
HADS scoring questionnaire.

Lower limb amputation is a commonly performed surgical procedure that is carried out for a variety of clinical reasons including peripheral vascular disease, diabetes, trauma and malignancy. The psychological reaction to the loss of a limb is complex and variable; depression, anxiety, as well as a wide range of other psychological responses are encountered [1]. Measuring the psychological effect of amputation is challenging and a variety of tools are available. The majority of these tools focus on quality of life or depression, such as the Patient Health Questionnaire (PHQ-9) [2]. The Hospital Anxiety and Depression scale (HADS) (Fig. 1), measures both anxiety and depression (HADS-A and HAD-D respectively) using seven questions for either metric and has been validated in multiple patient populations [3].

Through these psychological assessments, it has been demonstrated that there is a high prevalence of psychiatric morbidity existing among lower limb amputees with multiple risk factors identified [3]. The indication for amputation is important; higher rates of depression and anxiety demonstrated in patients undergoing surgical amputations compared those for trauma [1]. Postoperative outcomes such as pain also have an effect, with higher post-operative pain resulting in higher rates of depression and anxiety [4]. Socio-demographic factors also have an effect in the psychological adjustment to amputation [5].

There is a paucity of literature examining the effects that the level of lower limb amputation in non-traumatic amputations has on psychological health. Higher rates of anxiety have been demonstrated with traumatic trans-femoral amputations comparative to surgical amputations [5]. The aim of this prospective study is to evaluate the impact of amputation level on patients' mental health using the Hospital Anxiety and Depression Scale (HADS).

## 2 Methods

A prospective observational study was conducted between January 2021 and June 2021 in patients undergoing surgical major lower limb amputations at a single vascular unit in a tertiary referral centre.

Patients were identified from operating lists and after consent was obtained, patients were recruited into the study. Patients were suitable for inclusion to the study if they were undergoing a major lower limb amputation (trans-femoral or trans-tibial) and this was for a non-traumatic pathology. All traumatic amputations, revisions of amputation and minor amputations including toes or metatarsal amputations were excluded from the study. Patients were also excluded if they were unable to give informed consent or were unable to fill in or give answers to the questionnaire.

Patient demographics, duration of causative illness, medical co-morbidities and the functional status prior to the surgery was recorded from patient notes and electronic records. Length of hospital stay, discharge destination and readmission were also recorded. Participants were asked to fill in the Hospital Anxiety and Depression Score questionnaire within one week prior to surgery and six to eight weeks postoperatively either in the outpatient clinic or via telephone.

The collected data was analysed using Statistical Package for Social Science (IBM Corp. Released 2017. IBM SPSS Statistics for Windows, Version 25.0. Armonk, NY: IBM Corp). The work was fully compliant with the STROCSS 2021 criteria [6].

Ethical approval was not required as it was deemed as service evaluation. The study was registered with the local clinical audit department (CA_2021-22-102).

## 3 Results

We identified 82 patients, 49 patients met the inclusion criteria; 22 trans-femoral amputations (AKA) and 27 trans-tibial amputations (BKA). Baseline characteristics of patients in the two study groups are reported in Table 1.

**Table 1:** Patient demographics and characteristics in both groups.

		Type Of Intervention		Test of Significance
		AKA	BKA	
		Mean ± SD N (%)	Mean ± SD N (%)	P-Value
**Age**		69.9 ± 16.2	66.4 ± 11.1	8
**Sex**	**Male**	13 (59.1%)	21 (77.8%)	0.16^**C**^
	**Female**	9 (40.9%)	6 (22.2%)	
**Co-Morbidities**	DM	11 (50.0%)	17 (63.0%)	2
	HTN	11 (50.0%)	12 (44.4%)	0.70^**(C)**^
	IHD	7 (31.8%)	7 (25.9%)	0.65^**(C)**^
	AF	3 (13.6%)	3 (11.1%)	1.00^**(F)**^
	CVE	4 (18.2%)	4 (14.8%)	1.00^**(F)**^
	Kidney Disease	2 (9.1%)	9 (33.3%)	3
	COPD	4 (18.2%)	6 (22.2%)	1.00^**(F)**^
	Malignancies	4 (18.2%)	4 (14.8%)	1.00^**(F)**^
	Orthopaedic Surgery	4 (18.2%)	10 (37.0%)	0.15^**(C)**^
	Thromboembolic events	4 (18.2%)	4 (14.8%)	1.00^**(F)**^
	Previous Revascularization	11 (50.0%)	14 (51.8%)	0.90^**(C)**^
	Previous Amputations	8 (36.4%)	12 (44.4%)	0.57^**(C)**^
	Transfusion	9 (40.9%)	10 (37.0%)	
	Previous Psychological issues	4 (18.2%)	5 (18.5%)	1.00^**(F)**^
**Disability**	**Independent**	10 (45.4%)	16 (59.3%)	0.34^**(C)**^
	**Dependent**	12 (54.5%)	11 (40.7%)	
**Life style factors**	**Smoking**	12 (54.5%)	19 (70.4%)	
	**Alcohol**	5 (22.7%)	8 (29.6%)	0.59^**(C)**^

The study population was largely elderly, diabetic and male. The duration of causative illness varied widely between patients. Average hospital inpatient stay was long (mean 12.9 days). High rates of perioperative pain were found within the cohort (81.6%). Just over half of the patients were functionally independent prior to amputation (53.1%) and most were discharged to a rehabilitation facility (67.3%).

Perioperative HADS scores indicated a high degree of both anxiety and depression amongst this cohort, Table 2. Median anxiety scores in HADS-A demonstrated high levels of anxiety preoperatively, decreasing after amputation. Depression scores in HADS-D had less notable change although postoperatively the inverse trend was seen with higher depression scores.

**Table 2:** Pre-operative and post-operative HADS scores for all the cohort.

	Normal	Borderline	Abnormal	Marginal Homogeneity test
				P-Value
Depression Pre-operative	23 (46.9%)	12 (24.5%)	14 (28.6%)	0.01^**(M)**^
Depression Post-operative	16 (32.6%)	8 (16.3%)	25 (51.0%)	
Anxiety Pre-operative	10 (20.4%)	19 (38.8%)	20 (40.8%)	0.44^**(M)**^
Anxiety Postoperative	17 (34.7%)	12 (24.5%)	20 (40.8%)	

*Depression: a. Pre-operative < Post-operative (7).

b. Pre-operative > Post-operative (23).

c. Pre-operative = Post-operative (19).

*Anxiety: a. Pre-operative < Post-operative (18).

b. Pre-operative > Post-operative (12).

c. Pre-operative = Post-operative (19).

**Fig. 2:**
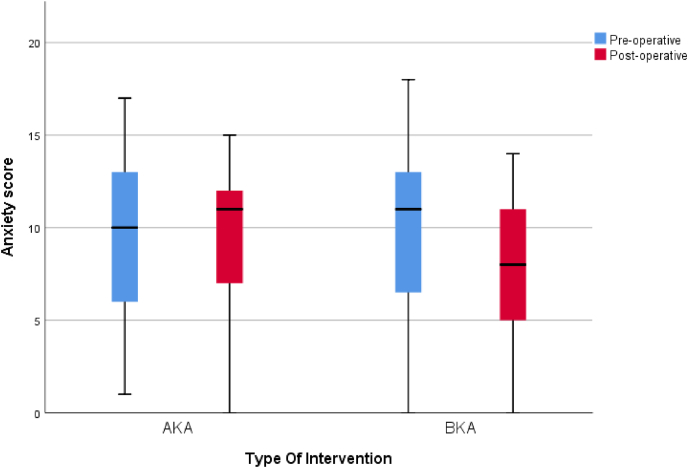
Comparison of the pre-operative and post-operative HADS-A in both groups.

**Fig. 3:**
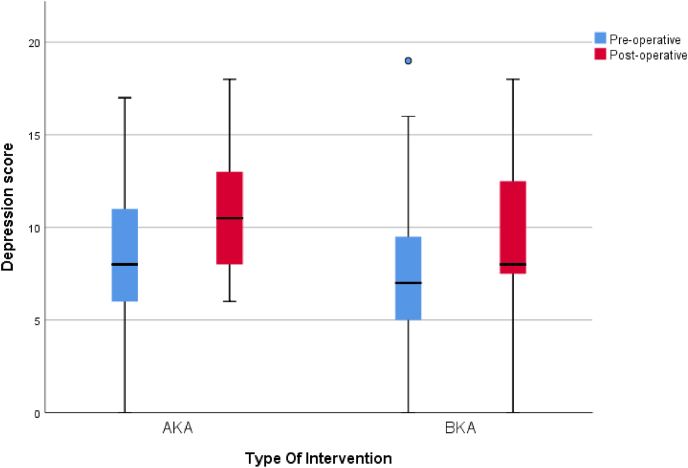
Comparison of the pre-operative and post-operative HADS-D in both groups.

Comparison between groups Table 1 showed no statistically significant difference in demographics and co-morbidities. Preoperative function was higher in the BKA group although this was not statistically significant. Preoperatively, higher rates of depression were noted in the AKA group comparative to the BKA group but no difference in anxiety although both results were not statistically significant, Table 3. Postoperatively, there was little change in the AKA group with high rates of anxiety and depression remaining and there was a trend to borderline depression scores from normal after amputation in the BKA group, Table 4. In the BKA group there was a statistically significant reduction in anxiety postoperatively (p < 0.05) Fig. 2, Fig. 3. Perioperative pain was found to be higher in the BKA group although this was also not statistically significant.

**Table 3:** Comparison of pre-operative anxiety and depression in both groups.

Pre-operative		Type Of Intervention		Test of significance
		AKA	BKA	
		Median (IQR) N (%)	Median (IQR) N (%)	P-Value
Depression		8 (6–11)	7 (5–10)	0.36^**(M)**^
Depression	Normal	8 (36.3%)	15 (55.6%)	0.21^**(C)**^
	Borderline	5 (22.7%)	7 (25.9%)	
	Abnormal	9 (40.9%)	5 (18.5%)	
Anxiety		10 (6–13)	11 (6–14)	0.91^**(M)**^
Anxiety	Normal	6 (27.3%)	10 (37.0%)	0.50^**(C)**^
	Borderline	5 (22.7%)	3 (11.1%)	
	Abnormal	11 (50.0%)	14 (51.8%)	

**(M)** Mann-Whitney test of significance.

**(C)** Chi-Square test of significance.

**Table 4:** Comparison of post-operative anxiety and depression in both groups.

Post-operative		Type Of Intervention		Test of significance
		AKA	BKA	
		Median (IQR) N (%)	Median (IQR) N (%)	P-Value
Depression		10.5 (8–13)	8 (7–13)	0.19^**(M)**^
Depression	Normal	3 (13.6%)	7 (25.9%)	0.41^**(C)**^
	Borderline	8 (36.4%)	11 (40.7%)	
	Abnormal	11 (50.0%)	9 (33.3%)	
Anxiety		11 (7–12)	8 (5–11)	0.07^**(M)**^
Anxiety	Normal	6 (27.3%)	11 (40.7%)	0.21^**(C)**^
	Borderline	4 (18.2%)	8 (29.6%)	
	Abnormal	12 (54.5%)	8 (29.6%)	

**(M)** Mann-Whitney test of significance.

**(C)** Chi-Square test of significance.

Total length of stay was noted to be longer in the AKA group (p < 0.05) although postoperative length of stay was similar. High rates of hospital re-admission were noted in both groups.

## 4 Discussion

Our results confirm the high rates of psychological morbidities demonstrated in the literature and that the HADS scoring system is a useful tool in measuring anxiety and depression in this cohort of patients [[1], [2], [3],^5^]. This study demonstrates high rates of preoperative anxiety in patients undergoing trans-tibial amputations and that this improves after amputation. This is contrary some of the evidence presented in the literature [7]. The finding may be due to the higher number of independent BKA patients and the higher expectation of functional capacity. The reduction may be due to the standard perioperative care in this unit including preoperative information leaflets and specialist amputation physiotherapist assessment. There is still clear improvements to be made in improving the psychological health of amputees however, as the large rates anxiety have been demonstrated not only in this study but also in the literature [7,8]. The high rates of psychological morbidity seen may also be a result of the high rates of perioperative pain identified within both groups. Pain has been demonstrated to be an important factor in the prevalence of psychological morbidity and quality of life and may explain the overall high HADS scores demonstrated in this study [4].

Two unusual results were also identified in this study; length of hospital stay and re-admission rate. Hospital stay was long for many patients and is likely influenced not only by the high co-morbidity burden these patients have but also the lack of community placements and rehabilitation beds in the United Kingdom caused by the global COVID-19 pandemic. Readmission rates were also exceptionally high compared to that described in the literature [9]. Unfortunately the data for the reason of readmission was not available but is again likely a result of the co-morbid nature of these patients which is supported by high morbidity and mortality rates described in the literature [10].

The limitations in this study include that it was performed in a single vascular unit and only a small number of patients were identified. The majority of patients were male; although this reflects a trend frequently seen in other studies, it does limit the generalizability of the findings. However despite this small sample, it is clear that a high degree of psychological co-morbidity exists. This remains a challenge given the multitude of factors that affect psychological wellbeing after amputation [4]. A multi-factorial approach including appropriate patient counselling, peri-operative pain control and mental health intervention in this population would appear wise; further research is required to determine the efficacy of these interventions.

## 5 Conclusion

In non-traumatic amputations, there appears to be a higher rate of pre-operative anxiety in patients undergoing trans-tibial amputation compared with trans-femoral amputees. However, the level of amputation does not appear to have a significant effect on psychological status of patients with high rates of depression and anxiety demonstrated in both groups.

## Ethical approval

Ethical approval was not required.

## Sources of funding

No source of funding.

## Author contribution

Ahmed Abdel Rahim (AA): data collection, statistical analysis, and wrote the paper.

Adam Tam (AT): Assisted in the literature search and Writing of paper.

Mathew Holmes (MH): writing the paper.

Devender Mittapalli (DM): team leading, conducting the research and final editing of writing.

## Trial registry number

The study is registered as an Audit project with the audit department in Derriford Hospital, University Hospitals Plymouth NHS trust.

## Guarantor

Ahmed Abdel Rahim.

## Consent

Ethics committee approval was not required as the study was done as an audit project.

The study was registered with the local clinical audit department (CA_2021-22-102).

University Hospitals Plymouth NHS trust.

## Provenance and peer review

Not commissioned, externally peer reviewed.

## Declaration of competing interest

No conflicts of interest.
